# Atomistic nonlinear carrier dynamics in Ge

**DOI:** 10.1038/s41598-023-32732-z

**Published:** 2023-04-06

**Authors:** Anshika Srivastava, Pankaj Srivastava, Anchal Srivastava, P. K. Saxena

**Affiliations:** 1 Tech Next Lab Inc., Lucknow, India; 2 Physics Department, University of Lucknow, Lucknow, India

**Keywords:** Engineering, Materials science, Mathematics and computing, Nanoscience and technology, Physics

## Abstract

An atomistic technique to successfully demonstrate the ultrafast carrier dynamics in Ge photoconductive samples is reported here. The technique is validated against the experimental findings and with the Drude conductivities. The impact of the various different scattering mechanisms is used to calibrate the experimental results. It is observed that the total scattering rate is not a constant parameter as contrast to Drude model which uses constant scattering rate as the fitting parameter to demonstrate the ultrafast carrier dynamics, but strongly dependent on the applied peak THz field strength. It also contradicts with the relaxation time approximation (RTA) method which uses scattering rate chosen on the empirical basis as the fitting parameter to demonstrate the ultrafast carrier dynamics. On the other hand the limitations and challenges offered by various types of density functional theories (DFT) pose lot of challenges. In current manuscript various types of scattering mechanisms i.e. acoustic, intervalley, Coulomb and impact ionization on the behavior of carrier conductivity are studied in details. The proposed technique has shown capability to extract low and high frequency conductivities accurately which is impossible through the Drude model or DFT based theories. It is observed that the free carrier absorption coefficient depends on the refractive index of the material at low THz frequencies. The solution of Boltzmann transport equation through Monte Carlo technique provides valuable insights for better understanding of ultrafast carrier transportation mechanism. The free carrier absorption spectra are found to be in good agreement with the experimental results at various THz field strengths.

## Introduction

Terahertz (THz) spectroscopy techniques are used for contact-less conductivity measurements of the metals, semiconductors, 2D materials, and superconductors^1–6^. However, the incapability to extract the carrier dynamics details in the bulk and nano- materials through THz probe pulses method is a serious drawback^1^. The widely used Drude model to fit the THz conductivities has shown incompetency to explain the suppression of the low-frequency conductivities which poses significant challenge especially for the case of nano-materials^6–9^. The other computational methods used to analyze the real-time electron thermalised carrier dynamics are based on density functional theory (DFT)^10,11^ and real time time-domain density functional theory (RT-TDDFT)^2,9,12^. However, the DFT based methods are computationally very expensive and can be applied to only small number of carriers. The treatment of long-range non-covalent interactions and exchange interactions in DFT based methods are also very tedious issue. Few ultrafast carrier dynamics studies are reported based on the solution of the Boltzmann transport equation (BTE) by Monte Carlo (MC) method in which relaxation time approximation (RTA) approach treated the carrier scattering rate as an empirical fitting parameter without any explanation^2,6,11–13^.

In current manuscript, the authors have attempted to address all the above mentioned issues in details through proposed innovative technique. The *TNL-TS™* simulator (a proprietary product from Tech Next Lab Inc.) is used to study the transport behavior of the hot carriers in photoconductive Ge film over the three energy valleys^14,15^. Using *TNL-TS™* simulator^16^, the solution of the BTE coupled with various nonlinear scattering mechanisms is carried out through Monte Carlo (MC) method. The contribution of acoustic, intervalley, Coulomb and impact ionization scattering mechanisms have given due consideration in the present study. The interaction of the THz photons with the electrons provides valuable information in terms of electron excitation and de-excitation processes through carrier population densities in the non-parabolic Γ, L and X valleys^17–21^.

## Technique

**Figure 1:**
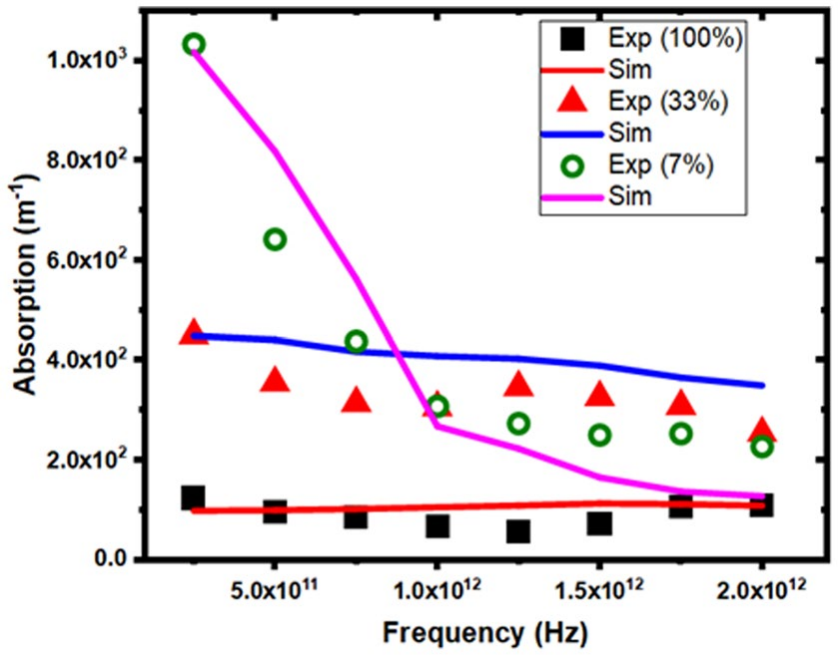
Absorption spectrum at peak THz intensity 1 (7%), 5 (33%), 15 (100%) MV/m with varying THz Frequencies. Data from present work is displayed as solid lines where as experimental results taken from reference^1^ are depicted by circles, triangles and squares respectively.

The proposed technique is applied for accurate prediction of the ultrafast carrier dynamics properties e.g. interaction of carriers with the THz photons. To simulate the free particle motion on the three valleys over full band structure, the free flight of the carrier is assumed to be terminated by instantaneous random scattering event. The solution of Boltzmann transport equation (BTE) through Monte Carlo (MC) method consists of generating random free flight times for each particle, choosing the types of scattering processes based on material specific properties, occurring at the end of the free flight. The scattering events change the final energy and momentum of the particle after the scattering mechanism following the Fermi Golden rule, and then repeating the same procedure for the next free flight. The sampling of the carrier motion at various time steps throughout the simulation allows for the statistical estimation of physically interesting quantities such as the carrier distribution function, the average carrier drift velocity under varying THz fields and frequencies strengths, the average energy of the particles, etc. By simulating an ensemble of particles, representative of the physical system of interest, the non-stationary time-dependent evolution of the electron distributions under the influence of a time-dependent driving force arises due to THz field. The particle picture is realized where the carrier motion is decomposed into free flights terminated by instantaneous collisions (Fig. 1).

**Figure 2:**
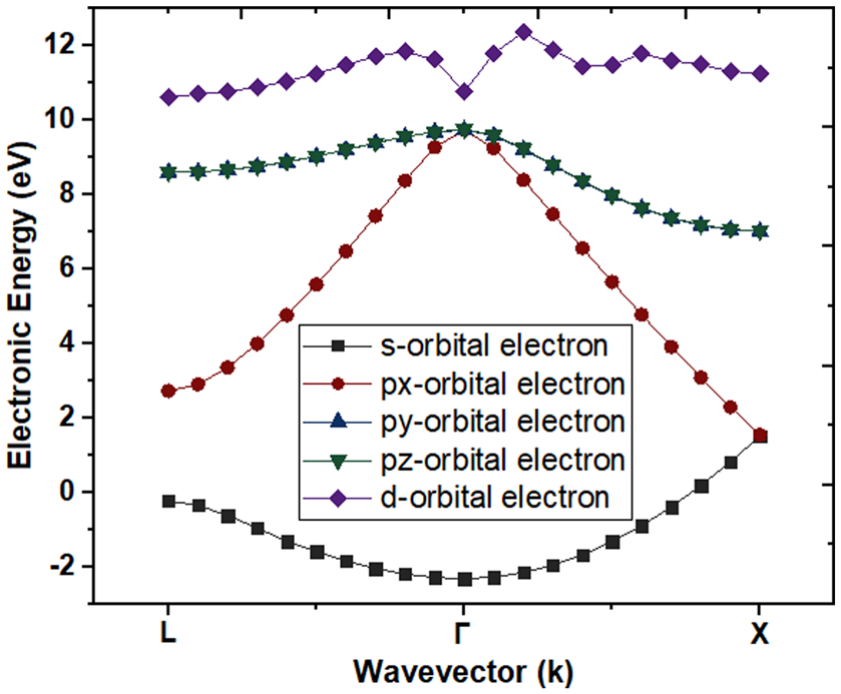
Three valley electronic band structure of Ge film depicting energy values at L-, Γ- and X-valleys.

The full electronic band structure of Ge (100) is essential requirement to extract various material parameters required for the electronic transportation. The thickness of Ge film is taken 100 nm. The *TNL-FB™* simulator^19^ is used to simulate full electronic band structure of Ge film under consideration as shown in Fig. 2. The E–k diagram depicts the variation of various energy levels associated with s-, p_x_-, p_y_-, p_z_- and first d- orbitals electrons at Γ, L and X valleys. More details of full electronic band structure simulation can be found in reference^19^. The various material parameters are extracted from the full band structure data (E–k) and tabulated in Table 1. Initially equal numbers of electrons are distributed in the non-parabolic Γ, L and X valleys under the steady state conditions. Most of the high energy level electrons i.e. X-valley electrons drop spontaneously to lower energy valleys i.e. at Γ and L valleys. The electrons are thermally populated (known as Boltzmann thermal population) in the L valley which is the lowest energy valley in case of Ge, whereas Γ valley lies in between X and L valleys.

**Table 1:** Ge Material Parameters used.

Parameters	Values	Parameters		Values
M (kg/m^3^)	5320	α (eV^−1^)	Γ	1
vs (m/s)	5400		L	4
Da (eV)	2.5		X	3
ε_*s*_	16.2	Equivalent valley	Γ	0
ε_∞_	16.2		L	0.3
*m*_Γ_	0.036		X	0
*m*_L_	0.455	*D*_*ij*_ (eV m^−1^)		2E10
m_X_	1.21	ℏω_*ij*_ (meV)		37
*L* − Γ (eV)	− 0.14	Doping (m^−3^)		2E21
*X* − Γ(eV)	0.635	Leff (µm)		0.6
ℏω_*LO*_ (meV)	0.23			

The Boltzmann transport equation (BTE) is characterized by a region of phase space about the points $(x, y, z, {p}_{x}, {p}_{y}, {p}_{z})$. The number of particles entering into this region in time *dt* is equal to the number of particles which were in the region of phase space (*x-v*_*x*_*dt, y-v*_*y*_*dt, z-v*_*z*_*dt, p*_*x*_*-F*_*x*_*dt, p*_*y*_*-F*_*y*_*dt, p*_*z*_*-F*_*z*_*dt*) at a time *dt* earlier. Here F represents the applied force field, ${v}_{i}$ is the carrier velocity and ${p}_{i}$ is the carrier momentum in the respective directions.

The change in the distribution function *df* which occurs during time *dt* due to the change in carrier’s wave vector in the coordinate space under the influence of the THz pulses.

1 $$\frac{df}{{dt}} = - {\varvec{v}} \cdot \nabla_{{\mathbf{r}}} f - {\varvec{F}} \cdot \nabla_{{\mathbf{p}}} f$$

Equation (1) represents only the change in the distribution function due to the motion of carriers in coordinate space and due to the momentum changes arising from the external THz force fields acting on the electrons. Electrons are also be transferred into or out of a given region in the phase space through the collisions or scattering interactions involving other particles of the distribution or scattering centers external to the assembly of particles under consideration. If the rate of change of the distribution function due to collisions, or scattering, is denoted by the function ${\left(\partial f/\partial t\right)}_{collision}$, the total rate of change of *f* becomes

2 $$\frac{df}{{dt}} = - {\varvec{v}} \cdot \nabla_{r} f - {\varvec{F}} \cdot \nabla_{p} f + \left. {\frac{\partial f}{{\partial t}}} \right|_{collision}$$

Rearranging Eq. (2),

3 $$\frac{df}{{dt}} + {\varvec{v}} \cdot \nabla_{r} f + {\varvec{F}} \cdot \nabla_{p} f = \left. {\frac{\partial f}{{\partial t}}} \right|_{collision}$$

Equation (3) represents the Boltzmann transport equation with the capabilities to handle the particle flow in the phase space.

The single-cycle THz pulses are introduced within the Ge film with a time delay of $3/4\mathrm{T}$, here T is the time period of each pulse. The delay in arrival of THz pulses at the sample is used to suppress the intrinsic carrier noise generated due to intervalley carrier transitions. The interaction of THz photons with the carriers results in form of the change in the energy and momentum associated with each carrier^16^.

The continuous time dependent THz pulse field is considered as^16^;

4 $$E\left( t \right) = E_{0} cos\left( {2\pi fpdt} \right)$$

Here, E_0_ is the peak field strength of the driving THz pulse, f is the THz frequency, p is the total number of time steps and dt is the time step. The interaction time (pdt) of THz pulses with carriers is selected in such a way that at least one complete wavelength of the driving frequency pulse passes through the sample during a single iteration refer to TNL-TS™ simulator’s manual^16^. The successive various mono-frequencies single cycle THz pulses interact with the carriers. The superposition of various mono-frequencies single cycle THz pulses can be used to generate the single THz pulse actual shape.

The total force field **F** is equal to the sum of the forces acting over the carriers due to the externally applied field and is given as

5 $$F = qE = \hbar \frac{dk}{{dt}}$$

Each carrier motion is assumed to consist of free flights terminated by instantaneous scattering events, which change the momentum and energy of the particle after scattering event within the limit of the Fermi Golden rule. The first task is to generate free flights of random time duration for each particle. This time duration is governed by total time of Monte-Carlo simulation of 20 ps which is divided in time steps *dt* = 0.4 fs giving N = 50,000 steps as a simulation point. The simulation initialize with 50,000 steps over a period of 20 ps. Hence, at least one period of the driving frequency is contained in every simulation wherein *f*_*THz*_ ≥ 0.1THz.

The change in carrier momentum as a function of THz field strength is obtained by integrating Eq. (5) to estimate the time evolution of carrier wavevector k between collision events as;

6 $${\text{k}}\left( {{\text{dt}}} \right) = k_{i} - \frac{{qE\left( t \right){\text{dt}}}}{\hbar }$$

Here, k_i_ is the wave vector at previous time step, ħ is Planck’s constant, q is the electronic charge and E(t) is the time domain THz pulse field strength.

The scattering rate, Γ[k(*t*)], the probability that a particle has not suffered a collision after a time *t* is given by ${e}^{\left[-{\int }_{0}^{t}\Gamma \left[k\left({t}^{^{\prime}}\right)\right]d{t}^{^{\prime}}\right]}$**.** Thus, the probability of scattering in the time interval *dt* after a free flight of time *t* may be written as the joint probability

7 $$P\left( t \right)dt = \Gamma \left[ {{\mathbf{k}}\left( t \right)} \right]\exp \left[ { - \int\limits_{0}^{t} {\Gamma \left[ {{\mathbf{k}}\left( {t^{\prime}} \right)} \right]dt^{\prime}} } \right]dt$$

The detailed Monte Carlo (MC) algorithm to solve coupled BTE with various scatterings is described in our previous publication^18^ and refers to reference^16^. For transient simulation, a synchronous ensemble of particles technique is used, in which the basic Monte Carlo algorithm is repeated for each particle in an ensemble representing the (usually larger) system of interest until the simulation is completed. Since there is rarely an identical correspondence between the number of simulated charges, and the number of actual particles in a system, each particle is really a *super-particle*, representing a finite number of real particles. The corresponding charge of the particle is weighted by this super-particle number. The super-particle number can be extracted through the algorithms implemented in TNL-TS™ (THz Spectroscopy) simulator.

The carrier drift velocity of each particle is recorded at each time step. The y-component of the carrier drift velocity at the p^th^ time step is given by;

8 $$v_{y,p} = v_{y,p - 1} + \frac{{eE_{0} dt}}{{m^{*} }}{\text{cos}}\left( {2\pi fpdt} \right)$$

Here, dt is time-step and ${v}_{y,p-1}$ is the carrier velocity at the previous time-step. The drift velocities of the carriers under transient conditions are computed by the ensemble average of velocity components as.

9 $$v_{d} \left( t \right) = \frac{1}{N}\mathop \sum \limits_{j = 1}^{N} v_{y}^{j} \left( {pdt} \right)$$

N is the total number of simulated particles and j labels the particular particle in the ensemble.

The time domain current density J(t) is calculated as

10 $$J\left( t \right) = qv_{d} \left( t \right)\left[ {N + n\alpha_{n} \left( E \right)L_{eff} } \right]$$

Here, q is the electronic charge, v_d_ is the carrier drift velocity and α_n_ is the impact ionization coefficient expressed as a function of THz field strength, L_eff_ is the characteristic length of the high field region^20–22^. N is the total number of carrier initiated and n is the number of carriers generated through impact ionization mechanism.

The frequency dependent complex conductivity is obtained by using Fourier transformation method refer to *TNL-TS™* simulator’s manual^16^,

11 $$\sigma \left( \omega \right) = \frac{\sum J\left( t \right)(\cos \omega t + i sin \omega t) }{{\sum E\left( t \right)(\cos \omega t + i sin \omega t) }}$$

The absorption coefficient depends on the conductivity,

12 $$\alpha \left( \omega \right) = \frac{{4\pi \sigma_{R} \left( \omega \right)}}{{n_{r} c}}$$

Here, c, ${\sigma }_{R}(\omega )$ and n_r_ are the speed of light, the real parts of conductivity and real part of refractive index respectively.

## Results and discussion

The section concentrates on the results obtained regarding specific aspects of hot carrier dynamics investigated using the technique proposed in current manuscript. The reliability of technique is verified against the experimental absorption spectra results and conductivities are compared against Drude model at various peak field strengths (1, 5, 15 MV/m) for Ge semiconductor. The strong contribution of various scattering mechanisms on the carrier dynamics provides valuable insight^24–29^. The details of various scattering mechanisms used in present work are given in online Appendix A.

Figure 1 depicts the comparison between the absorption spectra of n-type Ge thin film obtained through proposed technique and the experimental data taken from reference^1^ under similar conditions. Under the high THz field (15 MV/m) strength and frequency range 0.1–2THz, the simulated absorption spectrum matches with the experimental absorption spectrum, extremely well. At moderate peak field (5 MV/m) strength in frequency range (0.5–1.0 THz), a slight mismatch is observed, whereas it is almost matched with the experimental data in frequency range $1.0 < f_{THz} < 2.0$. A slight mismatch in frequency range $1.5 < f_{THz} < 2.0$ is also observed at the lower peak field (1 MV/m) strength. The slight mismatch at different peak field strengths (i.e. 1 MV/m & 5 MV/m) are attributed to difference in values of refractive indices used in present analysis. It is well known that the experimental refractive index of Ge semiconductor exhibits quadratic dependence on the wavelength of the externally impinging field^15,23^.The effect is known as THz-induced Kerr effect (TKE) and is found dominant under low to moderate field strengths with relation $n\left( \lambda \right) \propto 1/\lambda^{4}$^15^. In Eq. (12), the refractive index used for computation of absorption coefficient is taken independent of field and therefore a slight mismatch is observed in the absorption spectra curves obtained by the present model at low field strengths of 1 MV/m and 5 MV/m and with that obtained experimentally in Ref.^1^ under the same conditions. At high peak strength (15 MV/m) of THz field, the impact of Kerr effect is found to be negligible and the absorption spectrum matches well with the experimental one and also justify the experimental Kerr effect phenomenon. The self-focusing of the pulses occurs owing to the spatial variation of impact ionization at high peak strength (15 MV/m) of THz field. This phenomenon may be irrelevant to the Kerr effect^30–32^.

**Figure 3:**
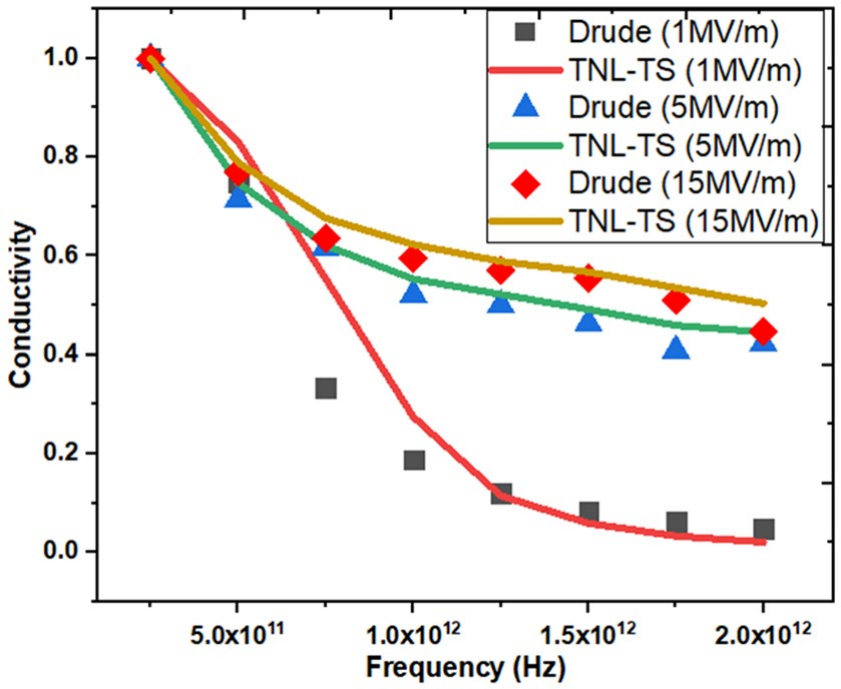
Complex conductivities at Peak THz Intensity 1, 5, 15 MV/m with ramping THz Frequencies. Conductivities extracted from the Monte Carlo simulations (solid lines) and symbols represent the conductivities fitted with Drude model^6^.

Figure 3 shows the comparison of complex conductivities derived here from the Monte Carlo (MC) based simulation technique, using Eq. (6) against the conductivity data fitted from the Drude model taken from reference^6^. In contrast to the assumption taken in reference^6^, it is observed that the total scattering time does not remain constant. The simulated conductivities obtained through simulation show excellent match with the conductivities obtained through Drude model along with accurate prediction of absorption spectra based on total scattering rate at different peak THz field strengths justifying the reliability of the proposed model. The contribution of various acoustic (electron–phonon), intervalley, Coulomb and impact ionization scattering mechanisms has observed to play significant role in deciding the saturation behavior of the conductivities at high field strengths.

**Figure 4:**
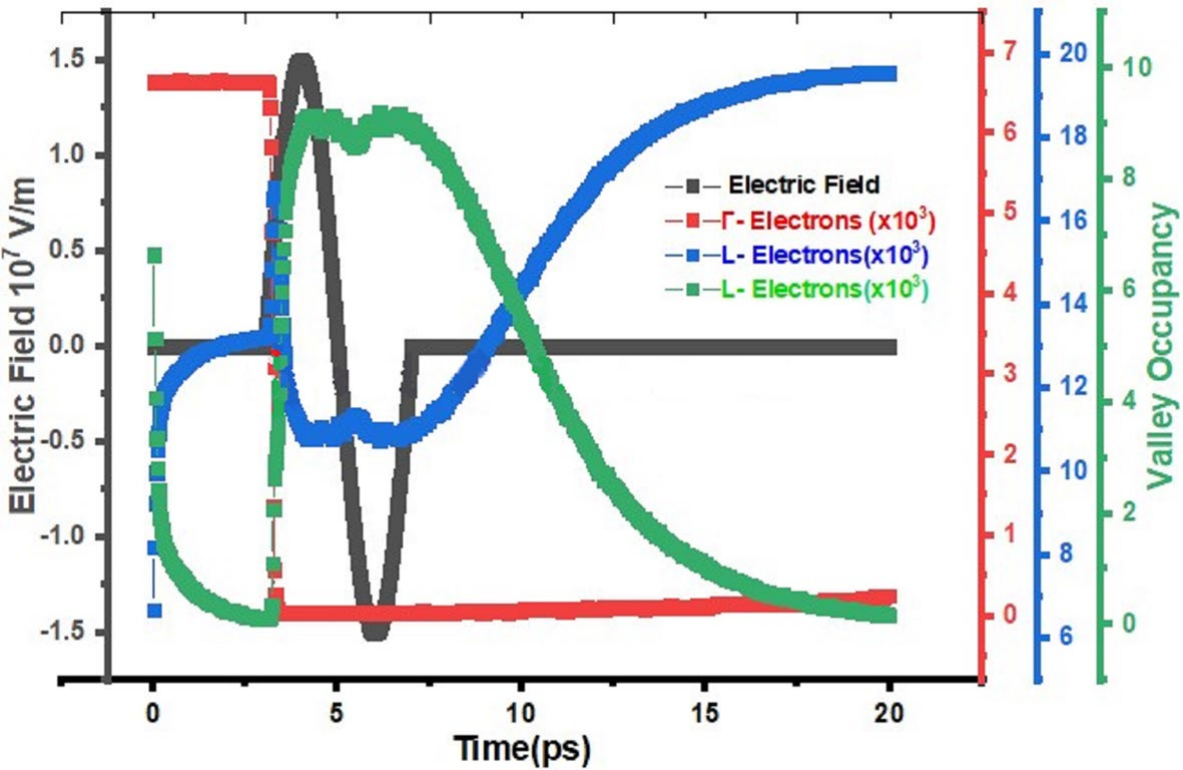
Variation in THz field Intensity with transition in number of electrons of Γ, L and X valley due to interaction of THz field at 0.25 THz frequency with respect to Time.

The variation of time-domain-carrier-occupancy densities at Γ-valley (Red), L-valley (Blue), X-valley (Green) with the impact of single cycle THz pulse (black) at f = 0.25 THz and E_0_ = 15 MV/m are shown in Fig. 4**.** The intrinsic noise due to the carrier transitions is suppressed by delaying arrival of each single cycle pulse with time delay of 3/4 T. Here, T is the time period of the pulse. Under steady state conditions, Boltzmann thermal population is observed in the L- and Γ-valleys. The interaction of single cycle THz pulse prompts the carrier intervalley transitions from L-valley and Γ-valley to X-valley, which is highest energy level in Ge. The variation in L- and X-valley population densities is attributed to the free carrier absorption mechanism. The interaction time of carriers with THz pulse is obviously dependent upon the pulse width. The free-carrier absorption mechanism can be explained with the help of valley carrier population densities at any instant during the simulation. Most of the X-valley electrons de-excite to lowest energy level (L-valley) as the single cycle pulse passed over through the sample and is clearly reflected from Fig. 4.

**Figure 5:**
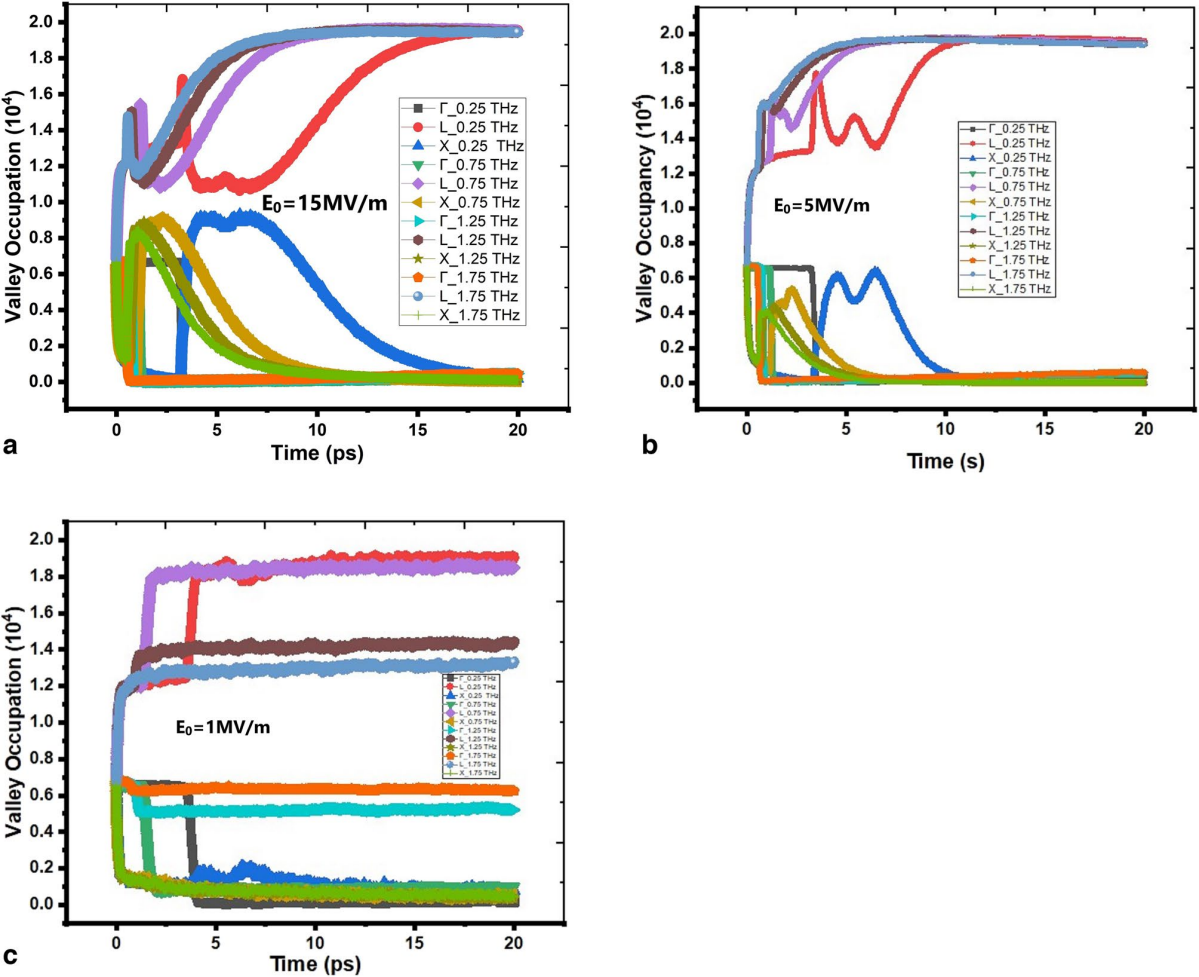
(**a**–**c**). Variation in Valley Population at various THz Frequencies at peak field intensity 15, 5 and 1 MV/m with respect Time respectively.

The Fig. 5a–c elaborate more details about the microscopic valley transitions behavior of the electrons with various single cycle THz pulses. The three valleys i.e. L-, Γ- and X-valley carrier population density at f = 0.25, 0.75, 1.25, 1.75 THz in Fig. 5a–c at peak field strengths E_0_ = 1, 5, 15 MV/m respectively reflect that the free carrier absorption mechanism is strongly dependent on the pulse width of each single cycle pulse. The carrier population of the three valleys changes in a different way with change in peak field intensities. The transfer of carriers from lower energy level (L-valley) to higher energy level (X-valley) increases with increasing field strength at a given frequency. Similar phenomena is observed in the transition behavior of carriers with increasing THz frequency at a given peak field strength. Such behaviors are expected too.

**Figure 6:**
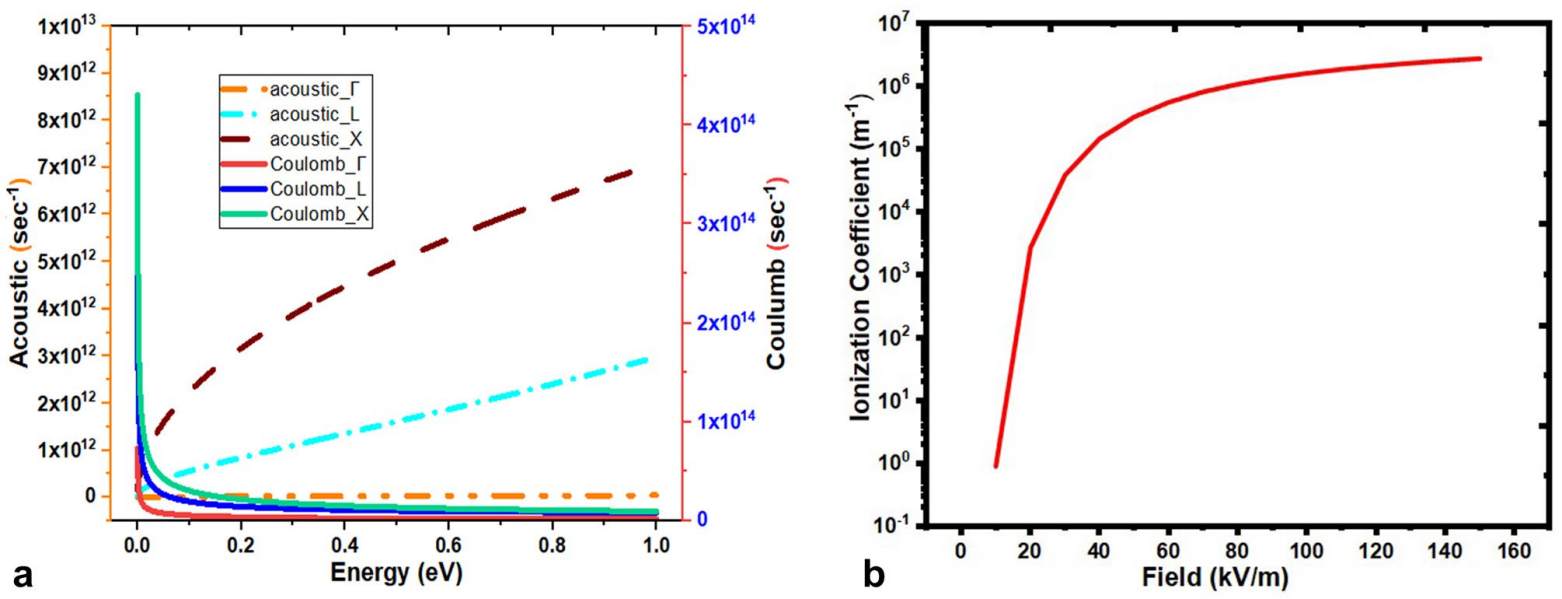
(**a**) Acoustic (electron–phonon) and Coulomb (carrier-carrier) scattering rate with respect to Energy (eV). (**b**). Variation of impact ionization coefficient with respect to varying THZ field intensity.

The acoustic and Coulomb scattering rates at L-, Γ- and X-valley are depicted in Fig. 6a. The Γ-valley acoustic and Coulomb scattering rates are very low as compared to L- and X-valley rates which indicate that the population in Γ-valley is very low. In the Ge semiconductor, Γ-valley lies at middle of L- and X-valleys. Under steady state conditions most of the free carriers remain at L-valley, the carrier interaction with single cycle THz pulses lead to excitation to X-valley due to free carrier absorption mechanism. The nature of acoustic and Coulomb scattering rates are opposite to each other at each valley and can be seen by the curves in Fig. 6a. The carriers gain higher vibrational energy and get transferred to X-valley and trapped there due to very high value of carrier effective masses; and the decrease in carrier population at L-valley results in constant acoustic scattering rate observed at L-valley. The Coulomb scattering is intrinsic scattering mechanism and applicable to the free carriers even in absence of THz pulses. The impact ionization mechanism generates more free carriers in the sample. At low frequency, the interaction time of carriers with THz pulses is more thereby leading to large number of transitions from L- to X-valley, therefore Coulomb scattering rate is high at X-valley. As the THz frequency increases, interaction time of carriers with THz pulses reduces and less number of transitions occurs. Therefore, the Coulomb scattering rate is observed almost constant in each valley under high THz frequency domain. It should be noted that impact ionization depends on peak field strength and not on the frequency of the pulse. The impact ionization coefficient is shown in Fig. 6b. The threshold field strength E_th_ for the initiation of impact ionization process in Ge is determined from Fig. 6b which is E_th_ ~ 12 kV/m, agrees well with that reported value of E_th_ in literature^20–22^. Here, the contribution to scattering due to holes generated along with electrons during impact ionization is not considered in present analysis. The increase in carrier density due to the impact ionization process increases Coulomb scattering rate resulting in degradation of the carrier conductivity at higher THz field strengths.

## Conclusion

In the present work, the ultrafast carrier dynamics based on various types of scattering mechanisms is demonstrated successfully. At low peak field intensity, the acoustic and intervalley scattering mechanisms are responsible for hot carriers drift and the resulting higher conductivities. The free carrier absorption is limited to intrinsic free carrier only. As the peak field strength increases beyond the threshold value 12 kV/m, the impact ionization process adds more free carriers and Coulomb scattering dominates over the other scattering mechanisms. Hence the probability of free carrier absorption process increases. Under high peak field strengths (5 MV/m & 15 MV/m) the saturation behavior of complex conductivity curves justify the dominant role of Coulomb scattering and free carrier absorption mechanism. The here proposed solution is capable to predict as well as explain the results in low and high frequency regime of THz spectroscopy experiments at atomistic scale.

## Supplementary Information

Supplementary Information.

## Data Availability

The data that support the findings of this study are available from the corresponding author upon reasonable request.
