# Supplementary material for: Atomistic nonlinear carrier dynamics in Ge
Source: Sci Rep. 2023 Apr 6;13:5630. doi: 10.1038/s41598-023-32732-z (PMC10079653; doi:10.1038/s41598-023-32732-z)
Supplement: Supplementary file 1 — Supplementary Information. [file 41598_2023_32732_MOESM1_ESM.docx]

**Appendix A**

An accurate comparison between experimental carrier mobility and theoretical calculation is of great importance for the determination of a variety of fundamental material parameters and carrier scattering mechanisms. The details of various carrier scattering mechanisms are used in current work is given as [24]-[29]

1. Acoustic Phonon scattering [24] –[29]

$\tau\left( E \right)=\frac{2\pi D_{ac}^{2}k_{B}T}{\hbar C_{l}}\left( \frac{{(2m_{d})}^{\frac{3}{2}}\sqrt{E(1+\alpha E)}}{4\pi^{2}\hbar^{3}} \right)\left( 1+2\alpha E \right)$ (A1)

1. Intervalley Phonon Scattering [24] –[29]

$\tau\left( E \right)=\left( \frac{\pi D_{ij}^{2}Z_{j}}{\rho W_{ij}} \right)*\left( n\left( W_{ij} \right)+\frac{1}{2}\mp\frac{1}{2} \right)*\left( \frac{{(2m_{d})}^{\frac{3}{2}}\sqrt{E_{f}(1+\alpha E_{f})}}{4\pi^{2}\hbar^{3}} \right)\left( 1+2\alpha E_{f} \right)$ (A2)

Here, $E_{f}=E\pm\hbar W_{ij}-\Delta E_{ij}$

1. Ionised Impurity Scattering [24] –[29]

$\tau\left( E \right)=\left[ \frac{\sqrt{2}e^{4}N_{l}m_{d}^{3/2}}{\pi\varepsilon_{s}^{2}\hbar^{4}} \right]*\left( \sqrt{E(1+\alpha E})*(1+2\alpha E \right)*\left[ \frac{1}{q_{D}^{2}\left( q_{D}^{2}+\frac{8m_{d}E(1+2\alpha E}{\hbar^{2}} \right)} \right]$ (A3)

Here $q_{D}=\sqrt{\frac{e^{3}N_{I}}{ℇk_{B}T}}$

1. Impact Ionization Scattering [20] – [22]

${1/\tau}_{ii}\left( E \right)=\left\{ \begin{aligned} \frac{{(E-E_{th}\left( T \right))}^{2}}{\tau_{p}\left( E \right)E_{th}^{2}(T)}; E>E_{th}(T) \\ \frac{{(E-E_{g}\left( T \right))}^{3.5}}{\tau_{p}\left( E \right)E_{g}^{3.5}(T)}; E_{g}<E<E_{th} \end{aligned} \right.$ (A4)

Here 1/τ_p_ is total phonon scattering rate, E is the carrier energy, $E_{th}$ is the temperature dependent threshold energy of impact ionization and is given as

$E_{th}\left( T \right)=\frac{E_{g}\left( T \right)}{E_{g}\left( 300K \right)}E_{th}(300K)$ (A5)

The ionization coefficient α_n_(E), is given by Chynoweth’s empirical formula;

$\alpha_{n}\left( E \right)=2.69\times{10}^{7}exp(-\frac{2.27\times{10}^{7}}{E})$ (A6)
